# A Combined One-Class SVM and Template-Matching Approach for User-Aided Human Fall Detection by Means of Floor Acoustic Features

**DOI:** 10.1155/2017/1512670

**Published:** 2017-05-30

**Authors:** Diego Droghini, Daniele Ferretti, Emanuele Principi, Stefano Squartini, Francesco Piazza

**Affiliations:** Department of Information Engineering, Università Politecnica delle Marche, Via Brecce Bianche, 60131 Ancona, Italy

## Abstract

The primary cause of injury-related death for the elders is represented by falls. The scientific community devoted them particular attention, since injuries can be limited by an early detection of the event. The solution proposed in this paper is based on a combined One-Class SVM (OCSVM) and template-matching classifier that discriminate human falls from nonfalls in a semisupervised framework. Acoustic signals are captured by means of a Floor Acoustic Sensor; then Mel-Frequency Cepstral Coefficients and Gaussian Mean Supervectors (GMSs) are extracted for the fall/nonfall discrimination. Here we propose a single-sensor two-stage user-aided approach: in the first stage, the OCSVM detects abnormal acoustic events. In the second, the template-matching classifier produces the final decision exploiting a set of template GMSs related to the events marked as false positives by the user. The performance of the algorithm has been evaluated on a corpus containing human falls and nonfall sounds. Compared to the OCSVM only approach, the proposed algorithm improves the performance by 10.14% in clean conditions and 4.84% in noisy conditions. Compared to Popescu and Mahnot (2009) the performance improvement is 19.96% in clean conditions and 8.08% in noisy conditions.

## 1. Introduction

The ageing of population is posing major concerns in governments and public institutions, since it will consistently increase the demand for healthcare services and the burden on healthcare systems [1]. The strategy adopted to reduce the impact of this demographic change on the society is to invest in intelligent technologies able to support the elderly directly in their homes [2].

Being the primary cause of injury-related death for the elders [3], human fall detection has been a major research topic in the last years. Several works appeared in the literature that present different solutions for a prompt detection of a human fall. The sensors at their basis are either “environmental” (e.g., infrared sensors, pressure, microphones, and cameras) if they are placed in the environment or “wearable” (e.g., accelerometers) if they are worn by the monitored person [4]. Regarding the algorithms, “analytical methods” classify an event as a fall or nonfall by thresholding the acquired signals or the features extracted from them [5]. These methods require manual tuning of their hyperparameters for different operating scenarios and subjects. On the contrary, “machine learning” methods learn to discriminate falls from nonfalls directly from the data [5]. They can be divided into “supervised methods,” which require a labelled dataset for training, and “unsupervised methods,” which base their decision on a normality model built from nonfall events only. Unsupervised methods have been proposed since human falls are “rare” events, and it would be difficult to capture a sufficient amount of examples for representing them in different operating scenarios (e.g., rooms, floor material) and subjects. Unsupervised methods, on the contrary, consider a human fall as an event that deviate from normality, and they are based on one-class classifiers. Their weakness is that certain events deviate from normality as the human fall (e.g., the fall of an object), and thus they may produce false alarms.

The approach proposed in this paper for reducing the problem consists of a combined One-Class Support Vector Machine (OCSVM) [6] and template-matching classifier that operate in cascade. The general idea is that a human fall produces a sound considerably different from the ones commonly occurring in a home (e.g., voices, sounds from electronic devices, and footsteps). The OCSVM is trained on a large set of “normal” sounds to detect acoustic events that deviate from normality. However, it is expected that certain acoustic events are as abnormal as a human fall (e.g., the fall of book and a chair), and thus they could raise false alarms. The template-matching classifier operates in a user-aided supervised manner and it is employed to reduce such errors by using a set of templates that represent these events. Templates are identified by the user that marks the occurrence of a false positive instead of a true human fall event. The fall detector operates on a environmental sensor, that is, on the signals captured by a Floor Acoustic Sensor (FAS), and it extracts Mel-Frequency Cepstral Coefficients (MFCCs) [7] and Gaussian Mean Supervectors (GMSs) [8] for classification by the OCSVM and template-matching classifier. The performance of the algorithm has been assessed on a large corpus of fall events created by the authors. The corpus contains human fall events reproduced by employing the “Rescue Randy” human-mimicking doll (https://www.simulaids.com/1475.htm) [9–11] and nonfall events represented by dropping of objects, music, and sounds related to common human activities. The experiments have been conducted in clean and noisy conditions in three scenarios: the first comprises human falls, human activity, and music; the second comprises human falls and object falls; the third represents the most realistic scenario and comprises all the classes of the first and second sets. The significance of the proposed method has been evaluated by implementing and assessing the algorithm with the OCSVM only and GMSs as input and the algorithm described in [12] based on OCSVM and with MFCCs as input.

The outline of the paper is the following: Section 2 presents an overview of the recent literature on fall detection algorithms based on environmental sensors.  Section 3 motivates the proposed approach and presents the contribution of the paper.  Section 4 describes the proposed fall detection algorithm.  Section 5 describes the experiments conducted to evaluate the performance of the approach. Finally, Section 6 concludes the paper and presents future developments.

## 2. Related Works

Fall detection approaches can be distinguished based on their sensing technologies and on the algorithm that discriminates falls from nonfalls [3, 13, 14]. As mentioned before, passive infrared sensors, vibration and pressure sensors, cameras, and microphones belong to the family of “environmental” sensors since they are located on the environment where the fall event takes place. On the contrary, accelerometers, heart rate, electrocardiogram (ECG), and body temperature sensors belong to the family of “wearable” sensors since they are embedded in a device worn by the monitored person.

The algorithms can be distinguished between “analytical methods,” which base their decision on thresholding the acquired signals or the related features, and machine learning methods that “learn” the characteristics of the fall signal directly from the data [5]. The methods proposed in [15–18] are “analytical methods” that employ wearable devices and decide whether a fall occurred or not by applying a decision threshold on the captured signals or on related features. The disadvantage of this solution is that it requires an a priori knowledge on the fall signal characteristics and manual tuning of the parameters of the algorithm, something that can be difficult to perform due to the variability of the operating conditions and of the subjects.

Machine learning techniques have, thus, been adopted in several recent works to overcome this drawback. Supervised approaches train the learning algorithm on a large dataset where all the classes of interest are represented. In [19], single-tree complex wavelet transform features are extracted from a floor vibration sensor and classification is performed by using a multiclass SVM. The training dataset comprises human falls, walking/running records, sitting on the floor, slammed door, and fallen book. Approaches based on audio signals are based on one or more microphones placed on the ceiling, on the walls, or on the floor. In previous works by some of the authors [20, 21], an acoustic sensor that operates similarly to stethoscopes has been employed to capture the acoustic waves that are transmitted through the floor. The algorithm is based on MFCCs and GMSs as features and on multiclass SVM trained on recordings of the falls of a human-mimicking doll and of several objects. In [22], the authors employed one aerial microphone and Perceptual Linear Predictive (PLP) coefficients as features. Classification is based on GMSs and SVM with a Kullback-Leibler divergence kernel that is trained to discriminate between falls and nine classes of nonfall events. In [23], the authors employed a circular array of eight microphones to determine the height of the sound source and to filter falls from nonfalls. MFCCs are used as features and the *k*-Nearest Neighbour (*k*-NN) classifier performs the final fall/nonfall discrimination. The classifier is trained on human falls and nonfall events comprising dropping of objects, walking, speech, and other sounds related to normal human activities. Li et al. [24] proposed a multichannel blind source separation technique based on Nonnegative Matrix Factorization (NMF). For additional ambient noise reduction a delay-and-sum beamformer has been used. Then, the MFCC features are extracted from the enhanced audio and finally a *k*-NN classifier is employed to discriminate the fall event from nonfalls. Differently, the system proposed in [25] captures the audio signal by using a smartphone placed on the table. Four different machine learning classifiers (*k*-NN, SVM, least square method, and neural network) are tested with four different types of features: spectrogram, MFCCs, linear predictive coding (LPC), and matching pursuit (MP). The best performance is achieved by using spectrogram features with ANN classifier with sensitivity, specificity, and accuracy all above 98%. Acoustic signals have been also employed in combination with signals acquired with different sensors. In [11], the authors combined features from sound and vibration sensors that are then employed by a naive Bayes classifier for classification. The experiments were conducted on a dataset containing falls of the “Rescue Randy” human-mimicking doll and four objects, and the resulting sensitivity and specificity were, respectively, 97.5% and 98.6%. Motion, sound, and video signals are employed in [26]. Signals are captured both from environment sensors and from body sensors. A fall is detected by analysing sounds and motion information, while visual and motion behaviour indicate the severity of the fall. The work by Toreyin and colleagues [27] combines PIRs, microphones, and vibration sensors. Signals are processed to extract features in the wavelet domain and HMM classifier is then employed to detect falls. The authors showed that using PIR signals 100% accuracy can be obtained. The approach proposed in [28] is based on video signals acquired from the cameras of Microsoft Kinect. The algorithm comprises a first stage where features are extracted from important joints of human skeleton and a second stage where an SVM is trained on the features extracted from the tracking of the joints.

The problem with supervised approaches is that they require that each class of interest is represented in the training dataset. However, with real human falls the variability of the environmental conditions and of the subjects makes it difficult or impossible to collect a sufficient number of examples that allow the algorithm to generalise well on unseen conditions [13]. Unsupervised approaches tackle the problem as a novelty detection task [29, 30], that is, by learning a normality model from data not related to human falls. Among approaches using wearable sensors, Zhou et al. [31] propose a fall detection algorithm based on activity transition extrapolated from accelerometers and gyroscopes. The main idea is to extract features from transition data between adjacent activities to recognise various kinds of normal and abnormal activities by means of an OCSVM. Popescu and Mahnot [12] evaluate three unsupervised methods for acoustic fall detection: Gaussian Mixture Models, nearest neighbour, and OCSVM. The acoustic signal is acquired with a single aerial microphone and the MFCCs contained in a window of 1 s are used for classification. The experiments are conducted on a dataset comprising falls and nonfalls represented by dropping objects, knocking, clapping, and sounds related to phone calls. A two microphones' approach has been presented in [32], where the algorithm first processes the stereo signal with a source separation stage to remove background noises. The classification algorithm is based on OCSVM and MFCCs as in [12]. In the dataset, normal events comprise sound originating from walking, bending, lying, and sitting. The authors did not consider falls of other objects that could significantly confuse the classifier; however they considered the presence of a television that produced the interfering sound. The results in terms of Area Under Curve are 0.9928 without interference and 0.9738 with 75% interference.

## 3. Motivation and Contribution

**Figure 1:**
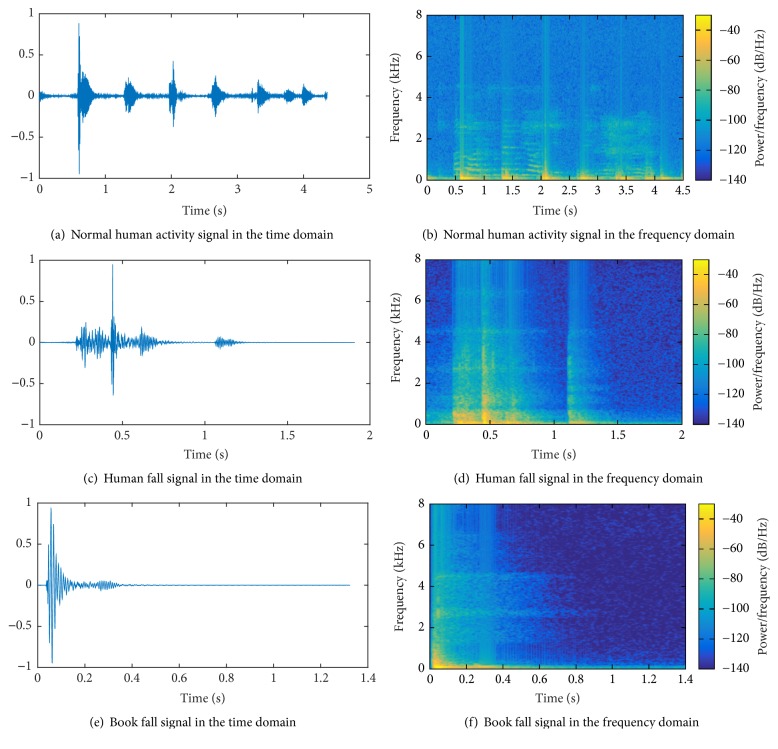
Time domain (on the left) and frequency domain (on the right) representation of a normal human activity signal (a-b), human fall signal (c-d), and book fall signal (e-f).

As shown in the previous section, “unsupervised methods” are able to overcome the need of manual tuning of “analytical methods” and the necessity of a large labelled dataset of “supervised methods.” In “unsupervised methods,” falls are discriminated from nonfalls based on a model of “normality” constructed from a large amount of nonfall events. However, certain events differ from the “normality” as human falls, and they may induce the classifier to produce false alarms. As an example, Figures 1(a) and 1(b) show, respectively, the waveform and the spectrogram of a segment of “normal” human activity (footsteps and speech). Figures 1(c) and 1(d) show the waveform and the spectrogram of a segment of human fall, and Figures 1(e) and 1(f) show the waveform and the spectrogram of a book fall. The figures show clearly that both falls signals differ significantly from the human activity one; thus a classifier may be induced to consider the fall of a book as the fall of a person.

The algorithm proposed in this paper reduces the problem by employing a multistage classification approach that combines a one-class classifier based on OCSVM with a template-matching stage. The OCSVM is trained unsupervisedly on a large corpus containing sounds that represent the “normality.” On the contrary, the template-matching stage employs a set of templates represented by a small number of feature vectors marked as false alarm by the user. Thus, robustness against possible false alarms is achieved by using only few examples of false positive classes without the need of multiple sensors. An additional advantage with respect to the state of the art is that the proposed approach is able to evolve and improve after its initial training, since the template set can be augmented as nonfalls events are detected. Finally, differently from the current literature [12, 22], the proposed approach employs Gaussian Mean Supervectors with OCSVM and captures the fall audio signal by means of a single Floor Acoustic Sensor.

## 4. The Proposed Approach

**Figure 2:**
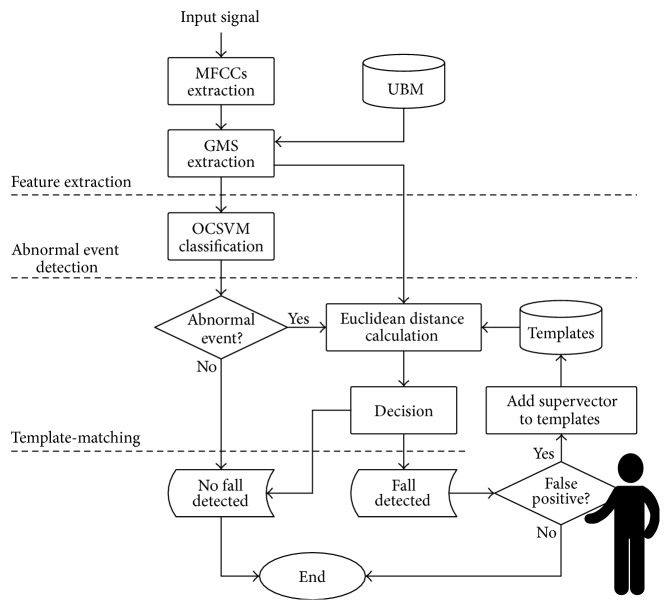
The block scheme of the proposed approach.

The proposed approach is composed of three stages (Figure 2): the first (“feature extraction”) extracts MFCCs from the input audio signal and then GMSs to describe the entire audio segment. The second stage (“abnormal event detection”) consists of a One-Class SVM classifier that discriminates between normal and abnormal sounds. To the authors' knowledge, OCSVM and GMSs have never been jointly used for acoustic fall detection. The third stage represents the innovative contribution of this paper for reducing false alarms in unsupervised approaches: it consists of a “template-matching” block that refines the output of the OCSVM and classifies the input data as fall or nonfall. The OCSVM is trained unsupervisedly on a large dataset of everyday sounds with the objective of discriminating normal from abnormal sounds. As mentioned before, the basic assumption is that the acoustic events related to human falls are “rare” with respect to sounds normally occurring inside a home. The template-matching stage, on the other side, requires a set of “template” instances that represent rare events that can be confused with a fall. Referring to Figure 2, the “template-matching” stage is composed of a set of “templates,” a block that calculates the distance between the input GMS and the templates (“Euclidean distance calculation”), and a “decision” block that decides whether the event is a fall or a nonfall by evaluating the magnitude of the distance. The rationale here is that certain acoustic events are as abnormal as falls and confuse the OCSVM: the template-matching stage reduces false positives by using a set of examples related to the most confusing classes. In this work, the algorithm is “user-aided”; that is, templates are indicated by the user each time the OCSVM produces a false positive. This is shown in Figure 2 with the person silhouette near the block that decides whether a detected fall is a false positive or not (“false positive?”). In general, however, it is possible to create the templates set a priori by recording several instances of possible false alarms events. Although rare, false alarm events (e.g., falls of objects) are certainly easier to reproduce in laboratory with respect to human falls. The remainder of this section describes the overall approach in detail, starting from the acoustic sensor employed for capturing falls sounds, the feature extraction stage, and the combined OCSVM/template-matching stages.

### 4.1. The Floor Acoustic Sensor

**Figure 3:**
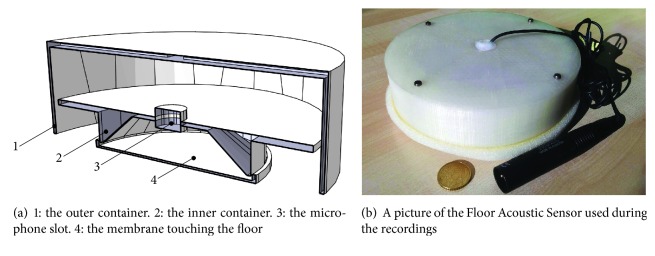
The Floor Acoustic Sensor scheme (a) and picture of the prototype (b).

The sensor employed to capture the sounds produced by a fall is shown in Figure 3: it is composed of a resonant enclosure and a microphone located inside. The acoustic coupling with the floor surface is guaranteed by a membrane that lays on it. As demonstrated by previous works by some of the authors [20, 21, 33], compared to microphones placed on walls or on the ceiling, this solution is better able to isolate the sounds produced by a fall from external interferences (e.g., voice, music). The enclosure has been manufactured in polylactic acid with a 3D printer, its diameter is 16.5 cm, and its height 5.5 cm.

Regarding the microphone, an AKG C 400 BL (http://www.akg.com/pro/p/c400-bl) has been inserted in the enclosure. The AKG C 400 BL is characterized by a hypercardiod directivity pattern; thus it has been oriented so that the maximum gain is towards floor.

### 4.2. Feature Extraction

#### 4.2.1. Mel-Frequency Cepstral Coefficients

**Figure 4:**
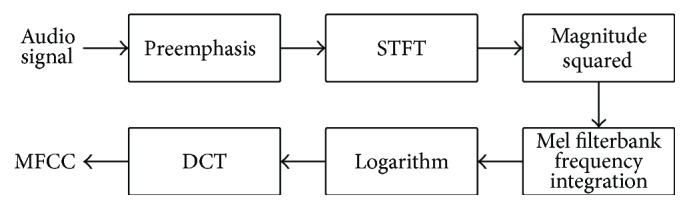
The MFCC feature extraction pipeline.

The feature extraction stage extracts low-level acoustic features represented by Mel-Frequency Cepstral Coefficients from the input audio signal. These are then employed to calculate Gaussian Mean Supervectors (GMSs), which represent higher level descriptors employed for the actual classification. MFCCs have been originally developed for speech recognition and speaker verification tasks; however they have been successfully exploited also for classifying falls [11, 20]. As shown in Figure 4, extracting MFCCs involves preemphasizing the input signal and filtering the output with a set of filters equally spaced in the mel space. After taking the logarithm of the energy in each band, the final coefficients are calculated by applying the Discrete Cosine Transform (DCT). In this work, preemphasis has not been applied, since the energy of the signals acquired with the FAS is concentrated at frequencies below 1 kHz and preemphasis would reduce the discriminative capabilities of the algorithm [20]. For further details on the MFCCs extraction procedure, please refer to [7, 20].

#### 4.2.2. Gaussian Mean Supervectors

**Figure 5:**
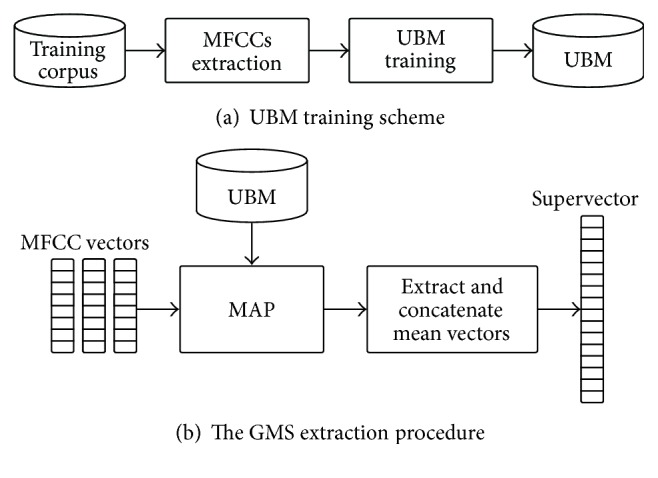
Training of the Universal Background Model from MFCCs (a) and extraction of Gaussian Mean Supervectors (b).

GMSs are higher level features composed of the means of a Gaussian mixture model (GMM) adapted with maximum a posteriori (MAP) algorithm [8, 34]. The GMM models a Universal Background Model (UBM) and is trained on a large set of audio data by using Expectation Maximization (EM) algorithm [35]. Then, a GMS is calculated by adapting the GMM with the MAP algorithm [36] and concatenating the adapted GMM mean values (Figure 5(b)).

More in detail, consider a sequence of *L* MFCC vectors **X** = {**x**_1_, **x**_2_,…, **x**_*L*_}, where each **x**_*l*_ has size *D* × 1. The GMM representing UBM is given by

(1) $$\begin{array}{c} p\left(\;x_{l}\;|\;\lambda \;\right)=\overset{J}{\underset{j=1}{\sum }}w_{j}p\left(\;x_{l}\;|\;\mu_{j},\Sigma_{j}\;\right), \end{array}$$

where *λ* = {*w*_*j*_, ***μ***_*j*_, Σ_*j*_∣*j* = 1,2,…, *J*}, *w*_*j*_ are the mixture weights, and *p*(·∣***μ***_*j*_, Σ_*j*_) is a multivariate Gaussian distribution with *D* × 1 mean vector ***μ***_*j*_ and *D* × *D* diagonal covariance matrix Σ_*j*_.

The GMS **M** of the sequence **X** is obtained by adapting the means of the UBM model with maximum a posteriori (MAP) algorithm and then concatenating the mean vectors:

(2) $$\begin{array}{c} M={\left[\;\mu_{1}^{T},\mu_{2}^{T},\ldots ,\mu_{J}^{T}\;\right]}^{T}, \end{array}$$

where *T* denotes the transpose operator. Regardless of the number of vectors in the sequence **X**, **M** is a *DJ* × 1 vector.

The number of Gaussians *J* can be determined on a validation set.

### 4.3. One-Class SVM

A One-Class SVM consists in a discriminant function that takes the value +1 in a small region that captures the majority of the data points of a set and −1 outside that region [6]. The discriminant function has the following expression:

(3) $$\begin{array}{c} f\left(\;x\;\right)=\mathrm{sgn}\left(\;\underset{i}{\sum }\alpha_{i}\cdot k\left(\;x_{i},x\;\right)-\rho \;\right), \end{array}$$

where **x**_*i*_ denotes the *i*-th support vector and *k*(·, ·) represents the kernel function, for example, the radial basis function *k*(**x**, **y**) = exp⁡(−*γ*‖**x** − **y**‖^2^). The position of the hyperplane, thus, defines the region that represents normal data points. For each point **x** that lies outside this region, the function *f*(**x**) takes the value −1, whereas, for point inside the region, it takes the value +1.

The terms *α*_*i*_ can be found by solving the solution to the dual problem:

(4) minα 12∑ijαiαjkxi,xj,subject  to   0≤αi≤1νl, ∑iαi=1.

The term *ν* ∈ 0,1] is a hyperparameter of the algorithm that is determined on a validation set.

The offset *ρ* can be obtained from the Karush-Kuhn-Tucker (KKT) condition with the expression [37]

(5) $$\begin{array}{c} \rho =\underset{j}{\sum }\alpha_{j}k\left(\;x_{j},x_{i}\;\right), \end{array}$$

which is satisfied for any *α*_*i*_ that is not at the upper or lower bound.

### 4.4. Template-Matching

The template-matching classifier operates on a set of templates, that is, supervectors, which can be defined a priori or selected by the user when the OCSVM detects an abnormal sound that is not a human fall. Denoting by **x** the supervector of the input signal and with *𝒴* = {**y**_1_,…, **y**_*N*_} the set of templates, the algorithm operates by calculating the Euclidean distance *D*^(*i*)^ = ‖**x** − **y**_*i*_‖ between the supervector to be classified and all the templates in the set. Indicating with *D*_min_ = min_*i*_⁡*D*^(*i*)^  , the supervector **x** is classified as a fall if *D*_min_ > *β* and as nonfall otherwise. The threshold *β* is a hyperparameter of the algorithm that can be determined on a validation set.

## 5. Experiments

### 5.1. Dataset

**Figure 6:**
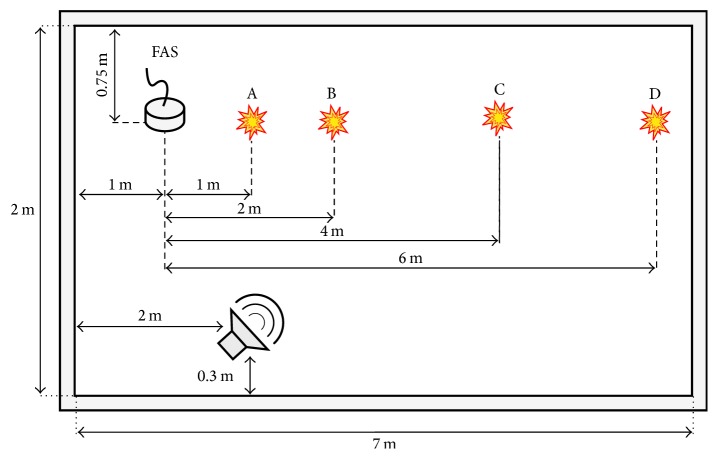
The recording setup: the letters A, B, C, and D indicate the positions of fall events.

The dataset (http://www.a3lab.dii.univpm.it/research/fasdataset) is composed of audio events corresponding to falls of humans, objects, sounds of normal activities (voices, footsteps, etc.), and music [20]. Acquisitions have been performed in a rectangular room measuring about 7 m × 2 m using a Presonus AudioBox  44VSL sound card and the FAS positioned on the floor (Figure 6).

Human falls have been simulated by means of “Rescue Randy,” a human-mimicking doll employed in water rescues. The doll has been dropped from upright position and from a chair, both forward and backward, for a total of 44 events, all included in the “human fall” class. Regarding falls of objects, a ball, a metal basket, a book, a metal fork, a plastic chair, and a bag have been used to reproduce sounds similar to human falls that could produce false detection. Each fall event has been performed at four distances from the FAS, that is, 1, 2, 4, and 6 m (Figure 6). Furthermore, for each distance, the basket and the chair have been overturned from their natural position, while the other objects have been dropped at two heights, that is, 0.5 m and 1 m. Normal activities sounds have been recorded while persons were performing common actions, such as walking, talking, and dragging chairs. Finally, three musical tracks have been played from a loudspeaker and acquired back with the FAS. The first track contained classical music (W. A. Mozart, “Piano Trio in C Major”), while the second (Led Zeppelin, “Dazed and Confused”) and the third (Led Zeppelin, “When the Levee Breaks”) contained rock music. Musical tracks and normal activities sounds have been divided into segments whose lengths have mean and standard deviation estimated from instances of fall events. In addition, they have been employed alone and to create noisy versions of human and object falls occurrences in order to assess the algorithm in presence of interferences. The total number of occurrences for each class is reported in Table 1.

Acquisitions have been originally performed with a sampling rate equal to 44.1 kHz and 32-bit depth [20]. In the experiments, signals have been downsampled to 8 kHz and the resolution has been reduced to 16 bits. The choice of the sampling frequency is motivated by the analysis performed in a previous work by the authors [20], where it was shown that the signals recorded with the FAS have the majority of the energy concentrated at frequencies below 1 kHz.

### 5.2. Experimental Setup

The dataset described previously has been divided into one set for training the UBM and the OCSVM and three sets for evaluating the performance.

Training has been performed on the set shown in Table 2 composed of 947 occurrences (1773s) of human activities, classical music, and rock music. The assessment of the algorithm has been performed on the following datasets:

- Set 1 (human fall and background sounds): this set comprises 44 examples of human fall sounds and 44 examples of human activity and music sounds (Table 3(a)).
- Set 2 (human fall and object fall sounds): this set comprises 44 examples of human fall sounds and 44 examples of object fall sounds (Table 4(a)).
- Set 3 (human fall, object fall, and background sounds): this set comprises 44 examples of human fall sounds, 22 examples of background sounds, and 22 examples of object fall sounds (Table 5(a)).

For each set, the data have been divided into four folds, each composed of 11 human falls and 11 nonfalls. Then, one fold has been used for estimating the hyperparameters of the algorithm and three have been used for calculating the performance. The final performance is calculated by using the cumulative true positives, false positives, and false negatives obtained by varying the test folds. The validation phase consisted in searching for the number of components of the UBM, the values of *ν* and *γ* of the OCSVM, and the value of the threshold *β* in the template-matching stage. The values assumed by these variables are summarised in Table 6. The method employed for the template-matching decision threshold is explained in Section 5.3.

All the aforementioned datasets require a set of templates for the template-matching stage of the algorithm. In the case of object falls, the set of templates has been created by classifying a set of 372 object falls with the OCSVM and selecting the occurrences misclassified as human falls. In the case of background sounds, the set of templates has been created by calculating the Euclidean distance between each occurrence of the development-set and each occurrence of a set of 470 background signals and then selecting the segment whose distance is minimum. Details on the templates sets are shown in Tables 3(b), 4(b), and 5(b), respectively, for “Set 1,” “Set 2,” and “Set 3.”

The proposed approach has been compared to the algorithm presented in [12] based on OCSVM. The same algorithm has also been employed in [32] with a multimicrophone acquisition setup and a source separation stage. As in [12], the audio signals are divided into windows of the same lengths, and the related MFCCs are used for training the OCSVM and for classification. In [12], 7 MFCCs were extracted from audio signals sampled at 20 kHz and the length of the window was set to 1 s. Here, the feature vectors are the same as the proposed approach; that is, they are composed of the first 13 MFCCs and their first and second derivatives. The same window length of [12] cannot be employed here, since the dataset used in this paper comprises signals with lengths less than 1 s. Thus, the length of the window corresponds to the duration of the shortest event in the dataset, and it is equal to 576 ms (71 frames). Windows are overlapped by 50%, and, as in [12], an event is classified as fall if at least two consecutive frames are classified as novelty by the OCSVM. The same grid search procedure of the proposed approach has been adopted to search for the optimal values of *ν* and *γ* of the OCSVM.

The performance has been evaluated in terms of *F*_1_-Measure calculated as

(6) $$\begin{array}{c} F_{1}\text{-Measure}=\frac{2\cdot tp}{2\cdot tp+fn+fp}, \end{array}$$

where *tp* is the number of correctly classified falls, *fn* is the number of falls misclassified as nonfalls, and *fp* is the number of nonfalls misclassified as falls.

### 5.3. Choice of the Template-Matching Decision Threshold

**Figure 7:**
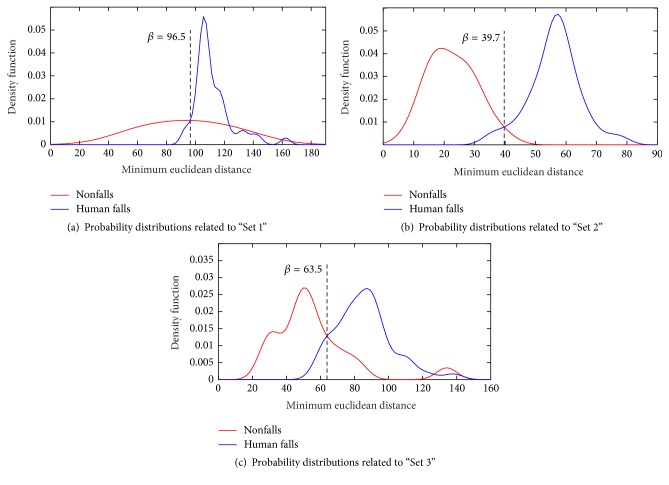
Probability distributions of the minimum Euclidean distances among the template sets and human falls and nonfalls in* clean* acoustic condition.

**Figure 8:**
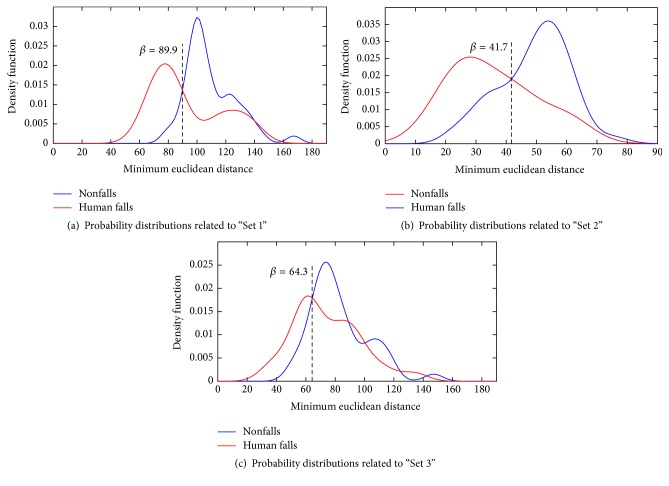
Probability distributions of the minimum Euclidean distances among the template sets and human falls and nonfalls in* noisy* acoustic condition.

A key point of the proposed approach is the decision threshold *β* in the template-matching stage. Choosing a too low value would result in a low number of false negatives and a high number of false positives. On the contrary, a too high value would result in a high number of false negatives and a low number of false positives. The choice of *β* has been performed by calculating the minimum Euclidean distance between each fall and nonfall event in the validation set and the set of templates. Figures 7 and 8 show, respectively, the probability distributions for the three sets in clean and noisy conditions. The decision threshold *β* has been chosen at the intersection point between the distribution of fall and nonfall distances. This choice represents a compromise that balances false positives and false negatives.

Observing clean condition distributions, in “Set 1” the two densities are considerably overlapped, while in “Set 2” the overlap is modest. It is expected that the possible improvement of the template-matching stage will be more consistent for “Set 2” with respect to “Set 1.” “Set 3” contains human activity and music occurrences as “Set 1” and object falls as “Set 2”: indeed, the probability distributions (Figure 7(c)) are more distinct with respect to the ones of “Set 1,” but not so much as the ones of “Set 2.”

Noisy condition distributions, shown in Figure 8, are in general less distinct compared to clean condition ones. The effect of being noisy is to flatten the distances of the fall and nonfall classes, thus resulting in less discriminative capabilities of the classifier. Thus, it is expected that the performance improvement in noisy conditions will be more modest with respect to the one obtained in clean condition.

### 5.4. Results and Discussion

**Figure 9:**
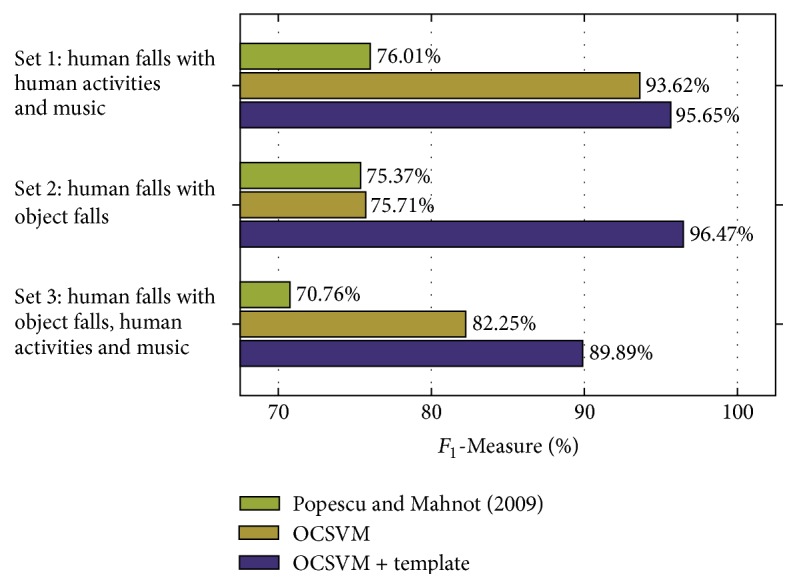
Results in* clean* conditions for the three test cases. “Set 1” comprises human falls, human activities, and music. “Set 2” comprises human falls and object falls. “Set 3” comprises human falls, object falls, human activities, and music.

Figure 9 shows the results in clean conditions obtained with and without the template-matching stage, respectively, denoted as “OCSVM + template-matching” and “OCSVM.” The results obtained with the method proposed in [12] are denoted with “Popescu and Mahnot (2009).” Observing the figure, it is evident that in all the three cases the template-matching approach is able to improve the performance with respect to “Popescu and Mahnot (2009)” [12] and the OCSVM only approach. In particular, in “Set 1,” which comprises human falls, human activities, and music, the performance improves by 2.03% with respect to OCSVM and by 19.64% with respect to “Popescu and Mahnot (2009).” This case can be considered as the least challenging of the three, since nonfalls events are considerably different from falls ones. Conversely, “Set 2” comprises both human falls and object falls, and thus it includes abnormal events whose pattern is similar to the one of human falls. Indeed, without the template-matching stage, the performance with respect to “Set 1” is 17.91% lower, mostly due the increased false positives rate that goes from 13.64% to 50.76%. The introduction of the template-matching stage considerably reduces the number of false positives, leading to an overall performance improvement of 20.76%. Regarding “Popescu and Mahnot (2009)” [12], the *F*_1_-Measure is below both OCSVM and the proposed approach; however it is less affected by the presence of object falls, since the *F*_1_-Measure decreases only by 0.64%. “Set 3” comprises human falls, human activities, music, and object falls and represents the most realistic test condition of the three. The result obtained by using the OCSVM classifier alone is 82.25%. As expected, this result is lower than “Set 1,” since object falls are also present, and higher than “Set 2,” since human activities and music segments are easier to discriminate. Introducing the template-matching stage, the performance improves by 7.64%, leading to an *F*_1_-Measure equal to 89.89%. Differently, the approach by Popescu and Mahnot [12] degrades by 5.25% with respect to “Set 1” and by 4.61% with respect to “Set 2,” demonstrating that it is less robust to the concurrent presence of object falls and daily human activities sounds.

**Figure 10:**
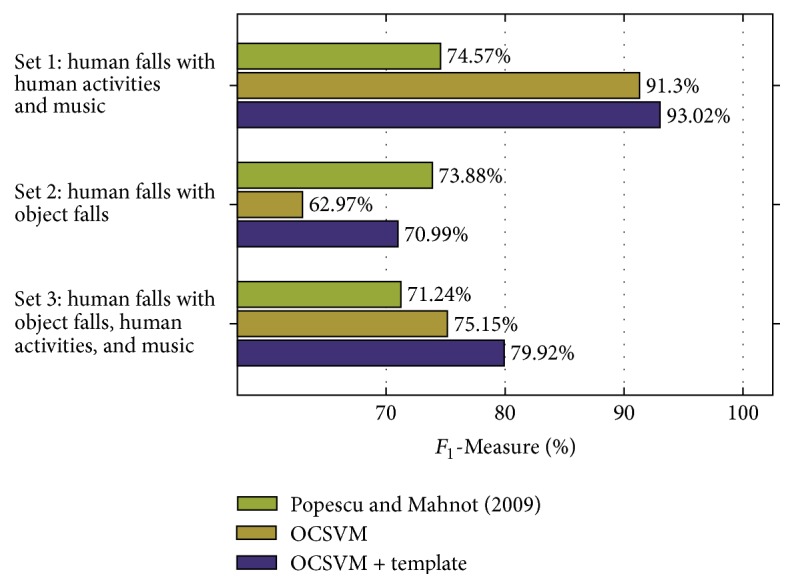
Results in noisy conditions for the three test cases. “Set 1” comprises human falls, human activities, and music. “Set 2” comprises human falls and object falls. “Set 3” comprises human falls, object falls, human activities, and music.

Figure 10 shows the results obtained for the three cases in noisy conditions. As expected, the performance decreases in all the three evaluated methods. In “Set 1,” the performance decrease is modest (2.32% for the OCSVM, 2.63% for the proposed approach, and 1.44% for “Popescu and Mahnot (2009)”), demonstrating that the OCSVM is able to effectively reject nonfall events corrupted by music interference. The use of the template-matching stage increases the performance by 1.72%, thus providing a significant improvement also in noisy conditions. In “Set 2,” the presence object falls corrupted by music significantly decreases the performance of the OCSVM, reducing the *F*_1_-Measure by 12.74% with respect to the clean “Set 2.” Template-matching provides a performance improvement of 8.02%, leading to an *F*_1_-Measure higher than 70%. The improvement is lower with respect to the clean “Set 2,” since the variability of the music interference makes the Euclidean distances of fall and nonfall classes more similar. The method by Popescu and Mahnot [12] achieves the highest *F*_1_-Measure in this case, confirming the good capabilities of rejecting dropping objects sound events observed in clean conditions. In “Set 3,” the proposed approach improves the performance by 4.77% with respect to OCSVM and by 8.68% with respect to “Popescu and Mahnot (2009),” confirming that it is able to achieve the highest performance in the most realistic scenario of the three.

In summary, the results demonstrated that the introduction of a template-matching stage significantly improves the performance both of the OCSVM only approach and of the method by Popescu and Mahnot [12]: averaging the results over “Set 1,” “Set 2,” and “Set 3,” the absolute improvement with respect to the former is 10.14% in clean conditions and 4.84% in noisy conditions. With respect to the latter [12] the improvement is 19.96% in clean conditions and 8.08% in noisy conditions. As shown in Figures 9 and 10, both in clean and in noisy conditions the *F*_1_-Measure of the method by Popescu and Mahnot [12] is close to 75% in “Set 1” and “Set 2” and close to 71% in “Set 3.” The different behaviour compared to the OCSVM only approach can be attributed firstly to the different feature representation of the audio signal (MFCCs instead of supervectors). Secondly, to the strategy adopted for classification: in [12], signals are divided into windows and a fall is detected if at least two consecutive windows are classified as fall. Differently, in the proposed algorithm, the overall signal is represented by a single supervector and classified as fall or nonfall.

Comparing the results in clean (Figure 9) and noisy (Figure 10) conditions, it is evident that techniques for reducing the impact of additive noise are needed. Additionally, the proposed solution requires the intervention of the user for selecting the templates after the first classification stage performed by the OCSVM. This aspect will be addressed in future works in order to make the algorithm completely unsupervised.

## 6. Conclusion

This paper proposed a combined OCSVM and template-matching classifier to discriminate human falls from nonfalls in a semisupervised framework. Fall signals are captured by means of a Floor Acoustic Sensor, and then MFCCs and GMSs are extracted from the acquired signal. The OCSVM discriminates between normal and abnormal acoustic events and the template-matching stage performs the final fall/nonfall decision. This stage employs a set of template supervectors represented by the events detected as abnormal by the OCSVM and marked as false positives by the user. The performance of the algorithm has been evaluated on a corpus containing human falls reproduced by a human-mimicking doll and nonfalls represented by sounds of falling objects, human activities, and music. In order to confirm the significance of the approach, it has been compared to the method proposed in [12] and to the OCSVM only approach. The results showed that, in the most realistic scenario, the proposed solution provides a performance improvement equal to 7.64% in clean conditions and equal to 4.77% in noisy conditions with respect to the OCSVM only approach and equal to 19.13% and to 8.68% with respect to [12].

In future works, the concurrent use of the FAS, aerial microphones, and heterogeneous sensors will be considered in order to further improve the robustness of the algorithm to external interferences. In addition, the possibility of removing the user from the classification loop will be explored, for example, by creating a set of templates related to object falls in different environments. Finally, in order to compare the proposed solution with approaches based on wearable devices, an appropriate corpus will be created where fall and nonfall events are recorded simultaneously with environmental and wearable sensors.

## Figures and Tables

**Table 1:** Composition of the dataset.

Class	Number of occurrences	Total length (s)
Basket	64	86
Fork	64	82
Ball	64	129
Book	64	63
Bag	64	57
Chair	96	157
Human falls	44	76
Human activity	665	1218
Music	776	1498

**Table 2:** Composition of the training-set.

Class	Number of occurrences	Total length (s)
Human activity	320	593
Music	627	1180

Total	947	1773

**Table:** (a) Composition of “Set 1”

Class	Number of occurrences
Human falls	44
Human activity	15
Music	29

**Table:** (b) Templates of “Set 1”

Class	Number of templates	
	Clean	Noisy
Human activity	13	11
Music	8	16

Total	21	27

**Table:** (a) Composition of “Set 2”

Class	Number of occurrences
Human falls	44
Basket	7
Fork	7
Ball	8
Book	7
Bag	8
Chair	7

**Table:** (b) Templates of “Set 2”

Class	Number of templates	
	Clean	Noisy
Basket	55	57
Fork	39	55
Ball	11	52
Book	26	57
Bag	26	56
Chair	86	89

Total	243	366

**Table:** (a) Composition of “Set 3”

Class	Number of occurrences
Human falls	44
Basket	3
Fork	4
Ball	4
Book	3
Bag	4
Chair	4
Human activity	8
Music	14

**Table:** (b) Templates of “Set 3”

Class	Number of templates	
	Clean	Noisy
Basket	52	57
Fork	57	57
Ball	19	55
Book	53	57
Bag	50	56
Chair	89	89
Human activity	11	4
Music	4	11

Total	335	386

**Table 6:** Hyperparameters of the algorithm and search space explored in the validation phase. The search space of the template-matching threshold *β* is not reported, since it is determined with the procedure described in Section 5.3.

Stage	Hyperparameter	Range
UBM	*J*	1,2, 4,…, 64

OCSVM	*ν*	0.1,0.2,…, 1.0
	*γ*	2^−15^, 2^−13^,…, 2^3^

Template-matching	*β*	See Section 5.3
